# Effectiveness of the aquatic physical therapy exercises to improve balance, gait, quality of life and reduce fall-related outcomes in healthy community-dwelling older adults: A systematic review and meta-analysis

**DOI:** 10.1371/journal.pone.0291193

**Published:** 2023-09-08

**Authors:** Renato S. Melo, Caroline Stefany Ferreira Cardeira, Damaris Scarleth A. Rezende, Vinícius J. Guimarães-do-Carmo, Andrea Lemos, Alberto Galvão de Moura-Filho

**Affiliations:** 1 Department of Physical Therapy, Universidade Federal de Pernambuco (UFPE), Recife, Pernambuco, Brazil; 2 Post-Graduate Program in Physical Therapy, Universidade Federal de Pernambuco (UFPE), Recife, Pernambuco, Brazil; 3 Department of Physical Therapy, Faculdade de Integração do Sertão (FIS), Serra Talhada, Pernambuco, Brazil; Prince Sattam bin Abdulaziz University, SAUDI ARABIA

## Abstract

**Background:**

Opting to use aquatic or land-based physical therapy exercises to improve balance, gait, quality of life and reduce fall-related outcomes in community-dwelling older adults (CDOAs) is still a questionable clinical decision for physiotherapists.

**Objective:**

Assess the quality of evidence from randomized or quasi-randomized controlled trials that used aquatic physical therapy exercises to improve balance, gait, quality of life and reduce fall-related outcomes in CDOAs.

**Methods:**

Articles were surveyed in the following databases: MEDLINE/PubMed, EMBASE, SCOPUS, LILACS, Web of Science, CENTRAL (Cochrane Central Register of Controlled Trials), PEDro, CINAHL, SciELO and Google Scholar, published in any language, up to July 31, 2023. Two independent reviewers extracted the data and assessed evidence quality. The risk of bias of the trials was evaluated by the Cochrane tool and evidence quality by GRADE approach. Review Manager software was used to conduct the meta-analyses.

**Results:**

3007 articles were identified in the searches, remaining 33 studies to be read in full, with 11 trials being eligible for this systematic review. The trials included presented low evidence quality for the balance, gait, quality of life and fear of falling. Land-based and aquatic physical therapy exercises improved the outcomes analyzed; however, aquatic physical therapy exercises were more effective in improving balance, gait, quality of life and reducing fear of falling in CDOAs. The meta-analysis showed that engaging in aquatic physical therapy exercises increases the functional reach, through of the anterior displacement of the center of pressure of CDOAs by 6.36cm, compared to land-based physical therapy exercises, assessed by the Functional Reach test: [CI:5.22 to 7.50], (p<0.00001), presenting low quality evidence.

**Conclusions:**

Aquatic physical therapy exercises are more effective than their land-based counterparts in enhancing balance, gait, quality of life and reducing the fear of falling in CDOAs. However, due to methodological limitations of the trials, this clinical decision remains inconclusive. It is suggested that new trials be conducted with greater methodological rigor, in order to provide high-quality evidence on the use of the aquatic physical therapy exercises to improve the outcomes analyzed in CDOAs.

## Introduction

Balance disorders show a prevalence of 68% among the population 65 years and older and are characterized by imbalance, gait problems, instability, nausea, dizziness, vertigo and frequent falls [1]. Falls are among the main causes of morbidity and mortality in community-dwelling older adults (CDOAs), and every year, one-third of these individuals suffer falls [2]. In addition, nearly half of older people over the age of 80 years have suffered falls and between one-fifth and one-third have sustained moderate or serious injuries, including fractures [2], which account for approximately 70% of accidental deaths in people over 75 years of age [3].

The main risk factors for falls in CDOAs are linked to aging and the decline in the following systems: musculoskeletal, sensory, cardiovascular and cognitive function, and changes in one or more of these systems have been associated with a greater risk of falling in CDOAS [4]. The prevalence of falls in CDOAs has increased due to aging of the world population, making falls prevention a significant challenge for health professionals, particularly physiotherapists [5].

According to the clinical fall prevention guidelines of both the American and British Geriatrics Societies [6], therapeutic exercise is a key factor in preventing falls in CDOAs. A therapeutic exercise program should include strength training, balance, gait and motor coordination, and studies with exercise programs longer than 12 weeks, involving 1–3 weekly sessions demonstrated the best results [6]. A decline in the risk of falling in CDOAs can be promoted by therapeutic exercise programs aimed specifically at rehabilitating balance and gait, with high frequency, duration and intensity [7].

Among the main therapeutic exercise programs used by physiotherapists to improve the balance and gait of CDOAs are land and water-based exercises. However, some CDOAs have difficulty performing land-based exercises involving balance, gait and motor coordination, due to fear of falling or by postural instability caused by the complexity of the motor task during exercise, which hinders their movement on land [8,9]. Aquatic physical therapy is an alternative to its land-based counterpart for these individuals, since the heated water, buoyancy and subsequent decrease in fear of falling enable these older adults to move more easily in the therapeutic swimming pool [8], promoting greater confidence, motor dexterity, range of motion and center of mass displacement.

Thus, similar to land-based physical therapy, aquatic physical therapy challenges the physical capacities of older adults, using the physical properties of water, such as hydrostatic pressure, buoyancy, viscosity and turbulence, to stimulate the tonic-postural reactions and balance of CDOAs [8]. While subjects stand in the water and maintain a stable upright stance over the base of support, water movement and turbulence overloading the postural control systems during standing, and reaching movement (while feet are fixed on the pools floor) and during change of support movement (e.g., stepping), this relative motion of water causing displacement of either the body’s center of mass (via water motion and turbulence) or in the base of support, thus challenging the postural control system and it continuously stimulates a reorganization of body balance stability [10], which can make the aquatic physical therapy exercises a differential in terms of rehabilitation of balance and gait in CDOAs.

Given the results that demonstrate the efficacy of both interventions in improving the balance, gait, quality of life and reduce fall-related outcomes in CDOAs [11–16], opting for aquatic or land-based physical therapy exercises to improve these outcomes has become a questionable clinical decision for physiotherapists. This is because no systematic reviews have assessed the evidence quality of these trials, and no meta-analyses have shown if these interventions to be equivalent, or whether one of them is superior to another in improving these outcomes in CDOAs, thereby justifying the present study. Thus, the aim of this systematic review was to assess the evidence quality of randomized or quasi-randomized controlled trials that used aquatic physical therapy exercises to improve balance, gait, quality of life and reduce fall-related outcomes in CDOAs.

## Methods

This systematic review was conducted in line with the Preferred Reporting Items for Systematic Reviews and Meta-Analysis (PRISMA) [17], and was previously registered in the PROSPERO, under number CRD42020191916 [18].

### Trials identification and selection

Ten electronic databases were used to search for trials: MEDLINE/PubMed, EMBASE, SCOPUS, LILACS, Web of Science, CENTRAL (Cochrane Central Register of Controlled Trials), PEDro, CINAHL, SciELO and Google Scholar. The last search was on July 31, 2023. There were no restrictions for time of publication or language and a manual search was conducted in the references contained in the selected articles in order to guarantee that relevant studies were included in this systematic review.

The search strategies used in the databases are shown in Appendix 1.

The articles found in each database were analyzed independently by each of the two reviewers (Rezende DSA and Guimarães-do-Carmo VJ), who judged their eligibility by title and abstract reading, according to the following inclusion criteria: randomized or quasi-randomized trials, including individuals over 60 years of age of either sex, with no physical problems, cognitive or neurological impairments, except vestibular dysfunction, and who walking without the need for assistive devices. The intervention had to consist of aquatic physical therapy exercises with at least one of the following outcomes: balance, gait, quality of life, fall-related outcomes, dizziness, vertigo, pallor and/or vomiting.

In the first analysis, the articles were divided into eligible or ineligible for this review. Articles whose abstracts could not clearly establish their eligibility or those with potential to be included in this systematic review, were selected for subsequent reading of the entire text. Disagreements regarding the inclusion or not of an article were resolved by the two reviewers and, for cases where no consensus was achieved, a third reviewer was asked to arbitrate (Melo RS).

For articles with a lack of information, the authors of the present review sent an email to the corresponding authors, in order to request the necessary information. It is important to underscore that we received answers from all the authors who were contacted requesting information to determine their inclusion or not in the systematic review.

### Methodological assessment of the trials

#### Assessment of the evidence quality and risk of bias

The evidence quality of the trials was assessed by the GRADE approach [19]. According to this tool, five items can interfere in the evidence quality of a clinical trial: risk of bias, inconsistency, indirectness, imprecision and publication bias. For each of these items, evidence was considered based on the following classification: not serious (no decrease in points), serious (decrease of 1 point) or very serious (decrease of 2 points), scored depending on the risk of bias contained in the trials.

For the GRADE risk of bias item, the Cochrane tool [20] was used to assess the risk of bias of the articles, analyzing the following stages: randomization, allocation concealment, blinding of volunteers and outcome assessors, lost or missing data, selective outcome description and others (if applicable). Each of these stages of the risk of bias instrument was assessed and the following classifications attributed: low risk of bias (green), unclear risk of bias (yellow) and high risk of bias (red), according to the biases present in the trials assessed.

#### Participants

The trials were included if the volunteers were CDOAs, of either sex, aged 60 years or older, with no physical problems, cognitive or neurological impairments, except vestibular dysfunction, and who walking without the need for assistive devices.

#### Interventions

The intervention group were treated with aquatic exercises managed/supervised exclusively by physical therapists, which stimulate balance and the vestibular system. Intervention control could have occurred with land-based physical therapy exercises, any other intervention or no intervention.

#### Outcomes assessed

The outcomes analyzed in this systematic review were divided into three categories: motor skills, clinical and otoneurological outcomes.

The motor skills assessed were balance and gait, which are also the primary outcomes of this review. Included were articles that evaluated balance based on the speed of center of pressure oscillation (anterior-posterior and mediolateral), or the area of center of pressure oscillation, assessed by a force platform or computerized dynamic posturography.

Also included were articles that investigated the balance of older adults using the following clinical tests or scales: Berg Balance Scale (BBS), Performance Oriented Mobility Assessment (POMA) Scale, Romberg Test, One Leg Standing Test, Functional Reach Test (FRT), or any other instrument used by the authors.

In relation to gait, articles that assessed any walking condition were included, such as gait speed, distance between feet, step width, or any other instrument that evaluates locomotion. Also included were studies that used the Timed Up and Go (TUG) Test, Dynamic Gait Index (DGI), accelerometers, camcorders, photos or materials such as paint to mark the footprints of older adults on the floor or on paper, and those that used talc or sand.

The clinical and otoneurological outcomes were the secondary outcomes of this review. The clinical outcomes were quality of life and fall-related outcomes. Quality of life could have been assessed by the SF-36 Questionnaire, or by the WHOQOL, or by any other instrument that the authors have used to assess quality of life. The fall-related outcomes were three: fear of falling, risk of falls and episodes of falls, respectively, evaluated by the instruments: Falls Efficacy Scale (FES), Activities-specific Balance Confidence (ABC), Fall Risk Screening Tool (FRST), Fall Risk Index (FRI) and self-reports or calendar/diary of falls. The otoneurological outcomes were dizziness, vertigo, pallor and vomiting, and trials that applied any instrument to measure these outcomes were considered, including the self-reports of the volunteers.

#### Data extraction and analysis

The data included in this review were extracted and recorded on a standardized form created by the authors. These data were entered independently into the Review Manager (RevMan) program, version 5.4, by both reviewers for subsequent verification of the information and discussion of possible discrepancies.

Data homogeneity was analyzed using the heterogeneity test; study data with a p-value of more than 0.05 were considered homogeneous and those with a heterogeneity index (I^2^) up to 30% were classified as having low heterogeneity. In the first analysis, a fixed-effect meta-analysis was considered; however, when methodological or clinical heterogeneities were observed in the studies, random-effect meta-analysis was selected. The meta-analyses were conducted in RevMan software.

## Results

### Flow of trials through the review

**Fig 1:**
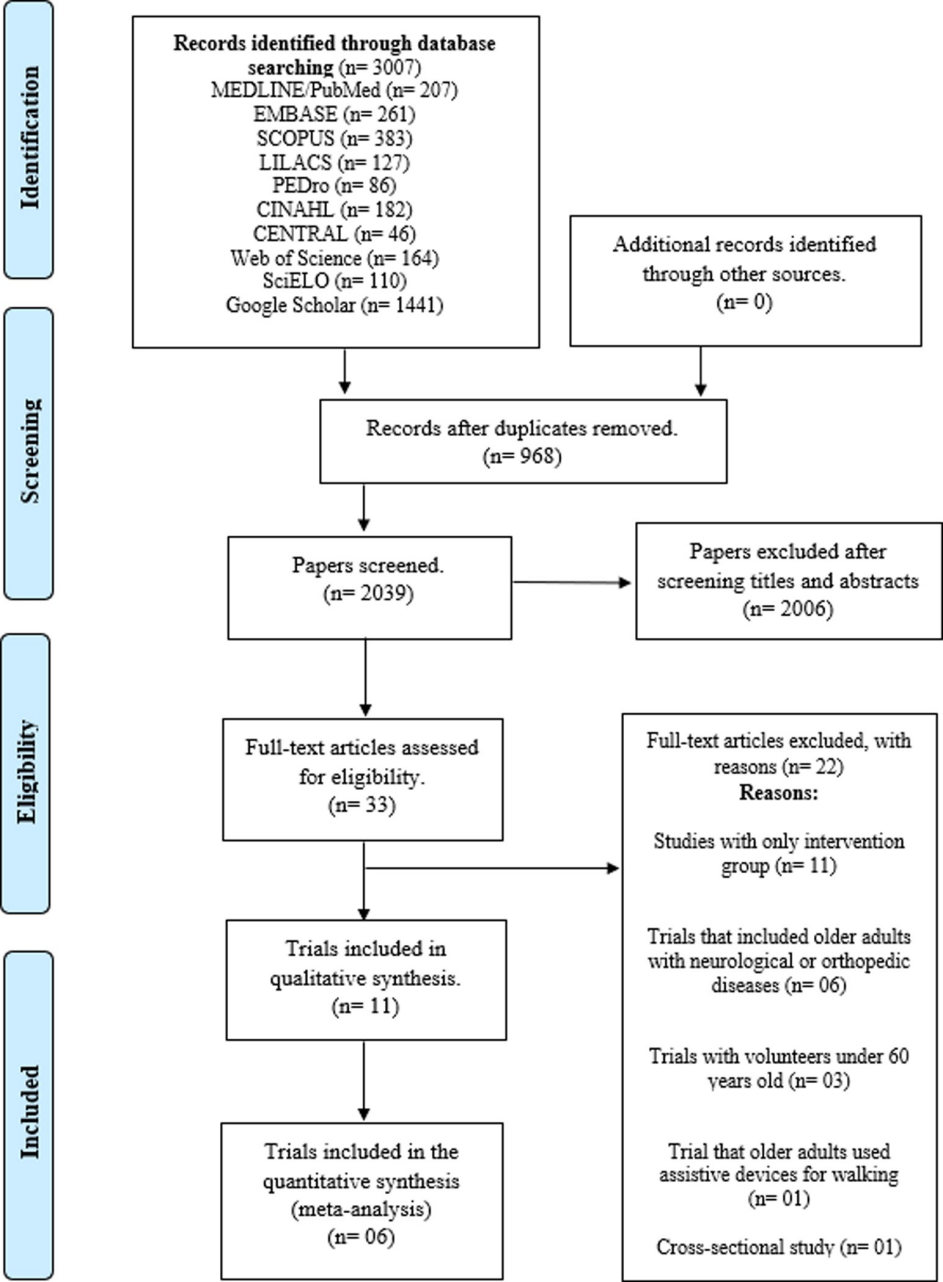
Flowchart of the studies analyzed in this systematic review, according to the Preferred Reporting Items for Systematic Reviews and Meta-Analyses (PRISMA).

A total of 3007 studies were identified, in line with search strategies, on the ten databases analyzed. After duplicate articles were removed, 2039 articles remained for analysis of titles and abstracts, 33 of which were read in their entirety. After reading, 11 trials [21–31] were considered eligible for the present review, nine of which are randomized [21,23,25–31] and two quasi-randomized [22,24]. Fig 1 shows the article selection flowchart, as recommended by PRISMA.

Of the excluded articles, eleven contained only the intervention group [14,32–41], six trials included volunteers with neurological and/or orthopedic diseases in their samples [42–47], three had individuals younger than 60 years [15,16,48], one trial included older adults that using walking aids device [49], and one was a cross-sectional study [13].

### Characteristics of the included trials

All eleven trials used aquatic physical therapy exercises for the intervention group and the control group was submitted in eight trials to land-based physical therapy exercises [21,23,24,26–30]. In three trials, the control group did not engage in exercises [22,23,31] and Elbar et al [25] conducted a crossover trial, where the intervention group started with aquatic physical therapy exercises and the control group performed none, reversing the interventions after 12 weeks. The characteristics of the trials analyzed are described in Tables 1 and 2.

**Table 1:** Summary of the included trials.

Authors	Country	Design	Characteristics of the Volunteers	Sample		Characteristics of the Interventions	
				CG	IG	CG	IG
Avelar et al [21]	Brazil	RCT	Community-dwelling older adults, of both sexes and aged 60 or over.	14	12	Muscle resistance training in the physical therapy gym, for 40 minutes, twice a week, for six weeks.	Muscle resistance training in the therapeutic pool, for 40 minutes, twice a week, for six weeks.
Bruni et al [22]	Brazil	Quasi-randomized trial	Old women, with clinical stability, age equal to or above 60 years and with independence for the gait.	13	11	No intervention.	Aquatic and land physical therapy exercises once a week, lasting 40 minutes, during ten weeks.
Cunha et al [23]	Brazil	RCT	Older adults, both sexes, aged over 60 and under 75, with no history of falls and who did not have difficulties for walking.	-	-	No intervention	Aquatic or land-based physical therapy exercises performed for 45 minutes, in 20 sessions, for 8 consecutive weeks.
Douris et al [24]	USA	Quasi-randomized trial	Older adults, both sexes, aged 65 and older who were independent ambulators and independent in activities of daily living.	05	06	Land physical therapy exercises were administered, for 20 to 30 minutes, 2 times a week, for 6 weeks.	Aquatic physical therapy exercises were administered, for 20 to 30 minutes, 2 times a week, for 6 weeks.
Elbar et al [25]	Israel	RCT	Healthy older adults, both sexes, age range 64–88 years, who ambulate independently.	17	17	No intervention for 12 weeks, and then they performed the same water exercise program as the intervention group.	24 sessions, for 40 min each session, twice a week over a period of 12 weeks, and then, they remained 12 weeks without receiving any intervention.
Franciulli et al [26]	Brazil	RCT	Older adults of both sexes, aged 60 or over, independent for walking, and with history of falls in the last 6 months.	06	08	Kinesiotherapy exercises performed on the soil, lasting 40 minutes, performed twice a week, for 2 months.	Aquatic physical therapy exercises, lasting 40 minutes, performed twice a week, for 2 months.
Oh et al [27]	South Korea	RCT	Community-dwelling older adults, both sexes, aged 65 or over and with history of falls in the last 3 months.	32	34	Land physical therapy exercises, for 60 minutes performed three times a week, for 10 weeks.	Aquatic physical therapy exercises, for 60 minutes performed three times a week, for 10 weeks.
Silva et al [28]	Brazil	RCT	Healthy older adults of both sexes, aged 60 or over, and with history of falls in the last 6 months.	19	16	Land physical therapy exercises, for 50 minutes, 2 times a week, for 10 weeks.	Aquatic exercise, for 50 minutes, 2 times a week, for 10 weeks.
Simmons et al [29]	USA	RCT	Older adults of both sexes, aged 65 or over and independent for walking and for activities of daily living.	12	10	Land physical therapy exercises were administered, for 45 minutes, twice per week, for 5 weeks.	Aquatic physical therapy exercises were administered, for 45 minutes, twice per week, for 5 weeks.
Tavares et al [30]	Brazil	RCT	Healthy and sedentary older adults of both sexes, aged 64 or over.	17	20	Land physical therapy exercises, for 60 minutes, twice a week, for 12 weeks.	Aquatic physical therapy exercises, for 60 minutes, twice a week, for 12 weeks.
Vale et al [31]	Brazil	RCT	Healthy and sedentary old women, aged 65–70 years.	26	26	No intervention for 16 weeks, and then they performed the same aquatic exercise program as the IG.	Aquatic physical therapy program consisted of 32 sessions, during 16 weeks and each session contained 60 minutes.

RCT: Randomized controlled trial; CG: Control group; IG: Intervention group.

**Table 2:** Methodological aspects and conclusions of the trials that used the aquatic and land physical therapy exercises to improve balance, gait, quality of life and reduce fear of falling in community-dwelling older adults.

Authors	Outcomes	Outcome measures	Instruments used for assessment	Control Group		Intervention Group		Conclusions
				Pre	Post	Pre	Post	
Avelar et al [21]	Balance and Gait	Functional Balance Gait-related Functional Tasks Dynamic Balance Gait Speed	Berg Balance Scale Dynamic Gait Index Tandem Gait Chronometer	**—**	**—**	**—**	**—**	Both physical therapy exercise programs (Land or Aquatic) improved the balance and gait of the older adults.
Bruni et al [22]	Balance and Gait	Balance and Gait	Performance Oriented Mobility Assessment Scale	**Balance:** 35.0±2.08 **Gait:** 15.0±1.78	**Balance:** 33.5±2.43 **Gait:** 13.3±2.14	**Balance:** 35.5±2.01 **Gait:** 15.4±2.46	**Balance:** 38.0±0.89 **Gait:** 17.4±0.81	This work showed significant results both in improving balance and gait in the old women practicing aquatic physical therapy.
Cunha et al [23]	Balance Gait Quality of Life Falls	Functional Balance Balance and Gait Gait Speed Quality of Life Fear of Falling	Berg Balance Scale Performance Oriented Mobility Assessment Scale Timed Up and Go SF-36 Questionnaire Falls Efficacy Scale	**—**	**—**	**—**	**—**	Aquatic and land physical therapy exercises improved the balance, gait, quality of life and reducing risk of falls of the older adults.
Douris et al [24]	Balance	Functional Balance	Berg Balance Scale	—	—	—	—	Regardless of the treatment medium (aquatic or land), significant improvements were evidenced on the Berg Balance Scale post-test.
Elbar et al [25]	Balance	Balance Stability (Sway Area)	Force Platform	**EO:** 80.6±0.23 **EC:** 114.7±0.70	**EO:** 73.9±0.31 **EC:** 91.0±0.52	**EO:** 93.5±0.50 **EC:** 141.6±0.81	**EO:** 86.9±0.62 **EC:** 113.2±0.80	Aquatic physical therapy exercises provided better control of balance in up-right standing to the older adults.
Franciulli et al [26]	Balance and Gait	Functional Balance Gait Speed	Berg Balance Scale Timed Up and Go	**BBS:** 48.2±0.90 **TUG:** 15.1±4.80	**BBS:** 51.0±1.70 **TUG:** 13.0±2.30	**BBS:** 48.3±2.20 **TUG:** 15.0±3.20	**BBS:** 53.6±3.90 **TUG:** 12.4±0.80	Both interventions (aquatic or land) were effective in improving balance and gait in the older adults with a history of falls.
Oh et al [27]	Gait Quality of Life Falls	Gait Speed Quality of Life Fear of Falling	Timed Up and Go Test SF-36 Questionnaire Falls Efficacy Scale	**TUG:** 6.25±0.15 **GH:** 50.1±15.4 **FES:** 123.3±39.2	**TUG:** 5.83±0.75 **GH:** 57.9±13.9 **FES:** 133.2±13.3	**TUG:** 7.42±1.26 **GH:** 55.2±11.3 **FES:** 96.3±39.2	**TUG:** 5.52±0.65 **GH:** 63.0±14.4 **FES:** 117,3±23.4	Aquatic physical therapy exercises are beneficial to improve the quality of life, as well as physical performance, of community-dwelling older adults compared with land exercise.
Silva et al [28]	Balance and Gait	Balance (Stability Limits) Gait Speed	Functional Reach Test Timed Up and Go	**FRT:** 20.8±1.80 **TUG:** 12.6±1.33	**FRT:** 27.6±1.80 **TUG:** 9.69±1.33	**FRT:** 22.2±1.85 **TUG:** 15.5±1.37	**FRT:** 34.1±1.85 **TUG:** 9.59±1.37	Aquatic and land physical therapy exercises showed to be greatly efficient, however aquatic exercises showed advantages, promoting more beneficial effects on balance and gait speed in older adults.
Simmons et al [29]	Balance	Balance (Stability Limits)	Functional Reach Test	23.1±2.80	28.7±3.80	21.6±5.30	34.0±4.10	Balance capabilities of the older adults were enhanced by the production of movement errors that was facilitated in a water environment.
Tavares et al [30]	Gait	Walking on level ground and close to home	Brazilian Version of OARS (Older American Resources and Services Questionnaire)	**WLG:** 4.70 **WCH:** 4.90	**WLG:** 5.60 **WCH:** 5.30	**WLG:** 5.10 **WCH:** 5.30	**WLG:** 5.10 **WCH:** 5.30	Aquatic and land exercises program significantly increases the ability of the older adults in walking on the level ground and to lie down and get out of bed.
Vale et al [31]	Balance	Functional Balance Balance	Berg Balance Scale Performance Oriented Mobility Assessment Scale	**BBS:** 53.3±1.20 **POMA:** 36.7±0.90	**BBS:** 53.1±1.50 **POMA:** 36.6±0.80	**BBS:** 53.4±1.80 **POMA:** 36.8±1.10	**BBS:** 54.9±1.20 **POMA:** 38.1±0.70	Sedentary lifestyle old women benefited from aquatic physical therapy exercise and improved the functional balance compared to a non-trained control group.

DGI: Dynamic Gait Index; BBS: Berg Balance Scale; TG: Tandem Gait; GV: Gait Velocity; POMA: Performance Oriented Mobility Assessment; TUG: Timed Up and Go; FRT: Functional Reach Test; FES: Falls Efficacy Scale; EO: Eyes open; EC: Eyes closed; GH: General Health; WLG: Walking on level ground; WCH: Walking close to home.

### Risk of bias

Nine of the eleven eligible trials mentioned randomization; however, only five [25–28,30] clearly described how this process took place. Two trials [22,24] exhibited high risk of bias, since they only reported that the sample was divided into control and intervention groups.

Only four of the trials used allocation concealment, via opaque envelopes [25,26,28,30], while seven made no mention of this procedure, indicating high risk of bias.

None of the trials reported sample blinding and only five controlled the blinding of outcome assessors [21,23,25,27,30]. In other words, in most of the studies, the examiners were aware of which group the older adults belonged to (control or intervention), suggesting high risk of bias for these trials.

Sample losses occurred in nine of the eleven trials; however, none of these conducted intention-to-treat analysis. One of the studies contained a selective description of the outcome, indicating high risk of bias for those with sample losses that did not carry out intention-to-treat analysis [21,23–29,31] and Cunha et al [23] selectively described the outcome data.

Another bias, identified as ‘other bias’ in Cochrane risk of bias tool was the difference between the average age of the older adults in both groups. Only two trials [25,31] showed no difference between the ages of the participants. The difference between the average ages of the subjects in eight studies ranged between 1–8 and 1–6 years younger in the intervention and control group, respectively. Cunha et al [23] did not provide the values of the average age of the groups.

**Fig 2:**
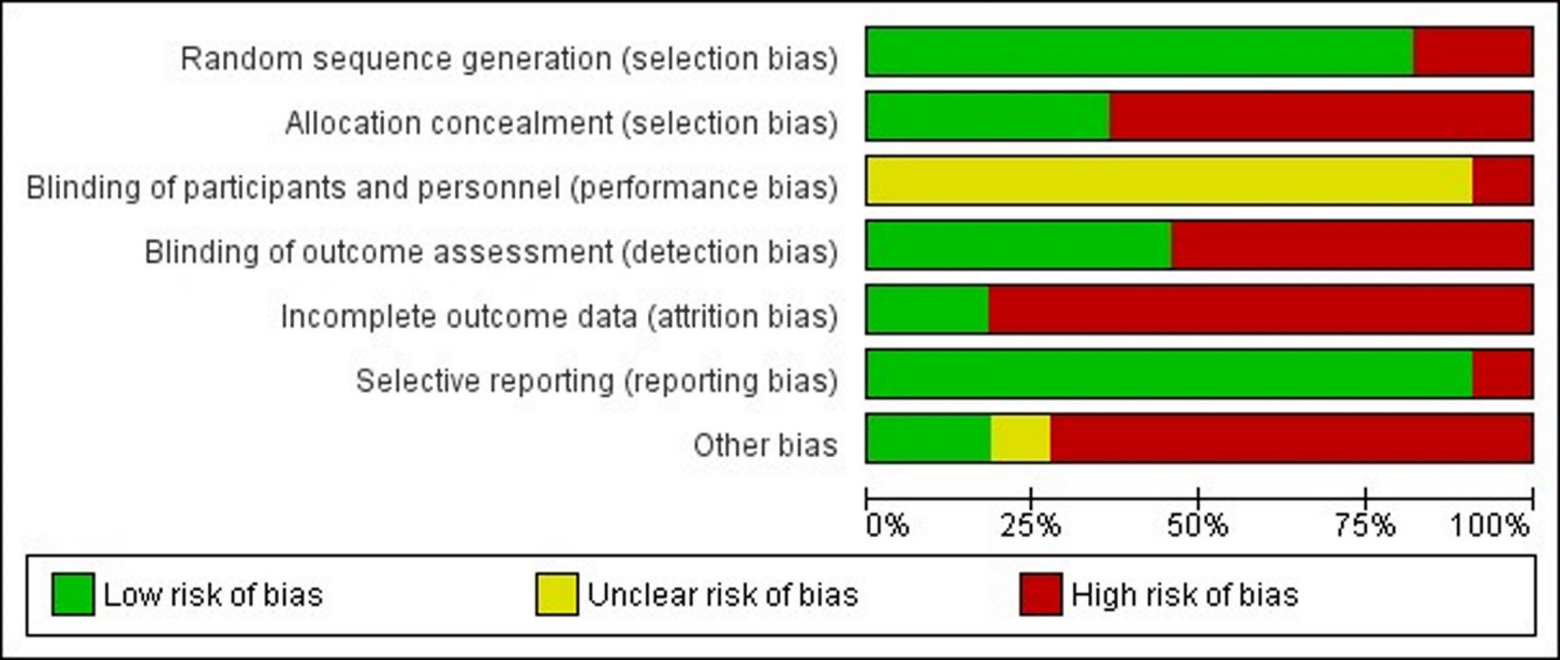
Risk of bias summary of the included trials assessed using the Cochrane risk of bias tool.

**Fig 3:**
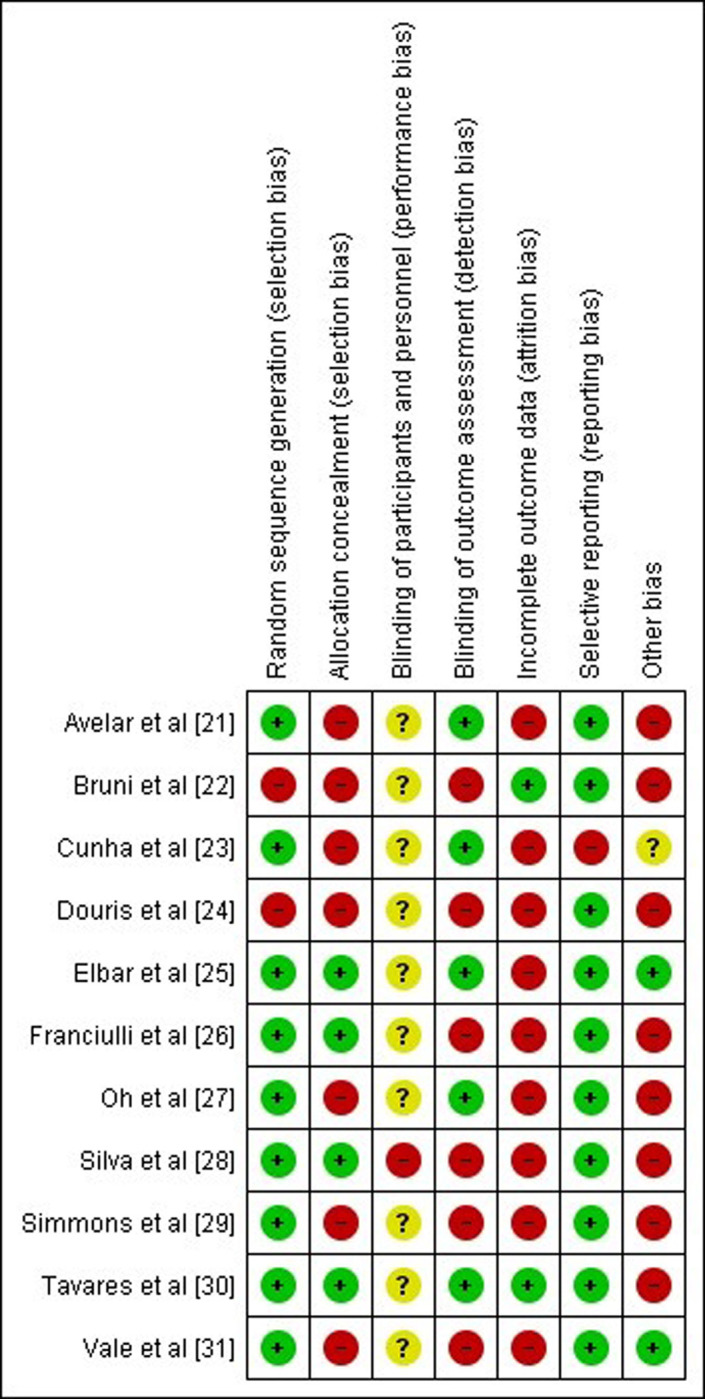
Risk of bias of each included trial assessed using the Cochrane risk of bias tool.

This difference between the average ages of the groups may under or overestimate the effect size of the interventions, thereby resulting in high risk of bias for the trials that displayed different average ages between the groups, which can be observed in Figs 2 and 3 and Table 3, illustrating the critical risk of bias analysis of the trials (grouped or separate) and the quality of GRADE evidence, respectively.

**Table 3:** Quality of evidence of the trials that used aquatic and land physical therapy exercises to improve balance, gait, quality of life and reduce fear of falling in community-dwelling older adults.

Quality assessment							∖ of patients		Effect		Quality	Importance
∖ of studies	Study design	Risk of bias	Inconsistency	Indirectness	Imprecision	Other considerations	Aquatic group	Control group	Relative (95% CI)	Absolute (95% CI)		
**Balance:** (follow up: mean 9 weeks; assessed with: Berg Balance Scale; Performance Oriented Mobility Assessment Scale; Functional Reach Test and Force Platform)												
09 [21–26,28,29,31]	RCT	very serious ^a,b,c,d,e^	not serious	not serious	not serious	none	108	113	-	-	⨁⨁◯◯ LOW	CRITICAL
**Gait:** (follow up: mean 10 weeks; assessed with: Dynamic Gait Index; Gait Tandem; Timed Up and Go Test; Performance Oriented Mobility Assessment Scale and Brazilian Version of Older American Resources and Services Questionnaire)												
06 [21,23,26–28,30]	RCT	very serious ^b,d,e^	not serious	not serious	not serious	none	84	81	-	-	⨁⨁◯◯ LOW	CRITICAL
**Quality of Life:** (follow up: 9 weeks; assessed with: SF-36 Questionnaire)												
02 [23,27]	RCT	very serious ^b,d,e,f^	not serious	not serious	not serious	none	34	32	-	-	⨁⨁◯◯ LOW	CRITICAL
**Fear of Falling:** (follow up: 9 weeks; assessed with: Falls Efficacy Scale)												
02 [23,27]	RCT	very serious ^b,d,e,f^	not serious	not serious	not serious	none	34	32	-	-	⨁⨁◯◯ LOW	CRITICAL

RCT: Randomized controlled trial; a: There was no random sequence generation; b: No allocation secrecy; c: There was no blinding of the evaluator of outcome; d: Loss or incomplete data without performing the intention-to-treat analysis; e: Comparison of older adult’s groups with disproportionate age groups; f: Trials in which there was selective description of the outcomes.

### Participants

The eleven trials included in this systematic review contained a total of 395 older adults and investigated whether aquatic physical therapy exercises are effective in improving balance, gait, and quality of life and reducing fall-related outcomes in this population. The intervention (aquatic physical therapy) and land-based or control (did not perform exercises) groups consisted of 168 and 180 CDOAs, respectively. Cunha et al [23] did not specify sample size in each group; however, 47 CDOAs remained at the end of the trial. We emailed the authors several times to obtain their data, but none of them responded.

### Interventions

The interventions contained similar therapeutic exercise programs, favoring meta-analyses. The therapeutic exercises performed by both groups were based on strength, muscle stretching, balance, gait and motor coordination, conducted in a therapeutic swimming pool or on land [21,26–30]. Only Douris et al [24] did not provide a detailed description of which therapeutic exercises were used in the interventions, reporting only that they were water and land-based.

### Outcome measures

Nine of the eleven trials assessed balance [21–26,28,29,31], seven gait [21–23,26–28,30] and two included the quality of life and fear of falling [23,27].

The trials used different instruments to assess the balance of the older adults. Five of the nine trials that evaluated balance used the BBS [21,23,24,26,31], three the POMA Scale [22,23,31], two the FRT [28,29] and one used a force platform [25]. It is important to underscore that some studies used more than one instrument to assess the balance of the older individuals in their samples.

In order to analyze gait, four studies used the TUG [23,26–28], two [22,23] the POMA Scale, one the Brazilian version of the Older American Resources and Services Questionnaire [30] and one assessed gait based on the DGI, Tandem Gait and gait speed, measured with a stopwatch [21].

Two articles evaluated quality of life and fear of falling and used the same instruments: the SF-36 Questionnaire (assessing all domains of this instrument) and Falls Efficacy Scale [23,27], respectively. The otoneurological outcomes (dizziness, vertigo, pallor and vomiting) were not included in any of the trials analyzed here.

### Meta-analyses

Three meta-analyses were conducted in this systematic review, two on the effects of aquatic physical therapy exercises on balance and one on gait.

**Fig 4:**
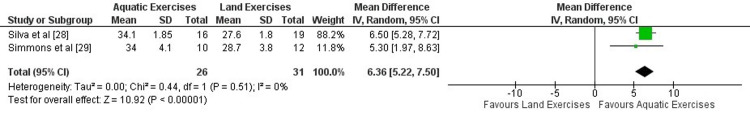
Comparison between the effects of the aquatic and land-based physical therapy exercises to improve balance of community-dwelling older adults, assessed by the functional reach test.

**Fig 5:**
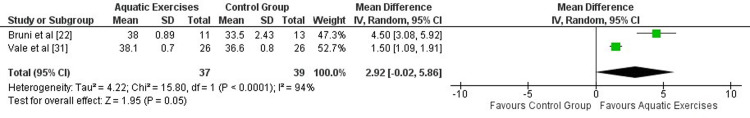
Comparison between the effects of the aquatic physical therapy exercises and not performing exercises to improve balance of community-dwelling older women, assessed by the performance oriented mobility assessment scale.

**Fig 6:**
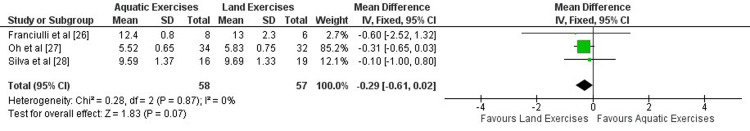
Comparison between the effects of the aquatic and land-based physical therapy exercises to improve functional mobility (gait speed) of community-dwelling older adults, assessed by the Timed Up and Go (TUG) test.

Meta-analyses on balance were performed with the data reported by Silva et al [28] and Simmons et al [29], who used the FRT, while Bruni et al [22] and Vale et al [31] assessed balance via the POMA Scale. The meta-analysis of functional mobility (gait speed) was carried out using the data of three trials: Franciulli et al [26], Oh et al [27] and Silva et al [28], who evaluated functional mobility using the Timed Up and Go (Figs 4–6).

Due to the differences between trials, in terms of session duration and total intervention time, we used the random effect to conduct the three meta-analyses.

A further meta-analysis could be performed in this review, on the balance outcome. The meta-analysis of balance, assessed by the BBS, could not be conducted due to the way the results were presented by the authors. Avelar et al [21] and Douris et al [24] published their data in figures but did not report the means and standard deviations of each outcome, and Cunha et al [23] provided the group means without the standard deviations. We emailed the authors to obtain these data, and Avelar et al [21] and Douris et al [24] replied that they no longer had this informations and after numerous attempts, Cunha et al [23] did not reply.

Two other trials also used the BBS to assess balance; however, Franciulli et al’s [26] sample was composed of CDOAs of both sexes and that of Vale et al [31] only older women, precluding a meta-analysis on the balance outcome assessed by the BBS in this review.

## Discussion

This is the first systematic review that assessed the evidence quality of the trials that used aquatic physical therapy exercises to enhance balance, gait, quality of life and reduce fall-related outcomes in CDOAs.

Eleven trials were analyzed and, although balance, gait and quality of life improved and fear of falling declined in CDOAs after the interventions, the quality of this evidence is low, due to the methodological limitations and biases present in the trials.

The main methodological limitations and biases observed were related to the three categories: biases in sample selection, methodological and traits of the older adults. As such, we decided to score and discuss them separately, as follows.

### Sample selection biases

Randomization was not reported by Bruni et al [22] and Douris et al [24] and four trials [21,23,29,31] failed to describe how it was conducted. This stage should be prioritized in clinical trials, since it guarantees intergroup homogeneity (intervention and control), thereby controlling selection bias. Another aspect absent in seven trials [21–24,27,29,31] was allocation concealment, a methodological process adopted to prevent researchers from knowing the group allocation of each volunteer beforehand.

The studies analyzed did not exhibit scientific rigor in these two stages, especially in allocation concealment, demonstrating that these sample selection biases should be better controlled in future trials on the topic, given that trials without allocation concealment overestimated the effect size of interventions by up to 30% [50].

### Methodological biases

Not controlling blinding of outcome assessors was another serious bias identified in six [22,24,26,28,29,31] of the eleven trials analyzed. Blinding of outcome assessors provides strong reliability of the findings presented by preventing prior knowledge of sample allocation from interfering in their response to treatment (conduction bias), or outcome assessment (detection bias). The lack of blinding of outcome assessors reduced the evidence quality of the trials, making their findings questionable, since studies that are not double-blind overestimate the size effect of interventions by 17% [50], demonstrating why there should be greater control of the blinding of outcome assessors in future trials.

Another bias observed in the trials analyzed was sample loss. Nine [21,23–29,31] of the eleven studies reported sample losses and none conducted intention-to-treat analysis. Intention-to-treat analysis allows all participants to be monitored until the end of the trial, irrespective of what occurs with some of them, thereby controlling sample loss bias. Excluding participants who did not remain until the end of the trial from statistical analysis may overestimate the effect size of the interventions, and intention-to-treat analysis aims at controlling this bias [51].

Another important limitation identified in the trials was comparison between interventions. Two trials [22,31] compared a group submitted to aquatic physical therapy exercises with one that performed no exercises, and when the aim of the trial is to analyze the effectiveness of a treatment, similar interventions are applied in terms of exercise characteristics, session duration, number of days per week and the total period, making these interventions comparable. This allows authors to conclude at the end of the trial whether the interventions are equivalent, or if any of them is superior in improving the outcomes studied, thereby helping in clinical decision-making.

When this does not occur and the trial compares the averages of the intervention and control groups, without submitting the latter to any intervention, it is expected that the intervention group will obtain the best post-treatment results, as observed in the two trials [22,31]. The absence of treatment for the control group makes it difficult to a clinical decision-making about the aquatic physical therapy exercises of these trials, representing a limitation in this regard for them.

Another trial [25] also did not compare simultaneous physical therapy exercises in water and on land. A crossover trial was conducted with the intervention group performing aquatic physical therapy exercises and the control group none. After twelve weeks, the control group initiated intervention with aquatic physical therapy exercises and the intervention group did not exercise for the next twelve weeks.

Thus, eight [21,23,24,26–30] of the eleven trials analyzed compared the effectiveness between aquatic and land-based physical therapy exercises. This comparison model should be maintained in future trials because it helps guide decision making and the clinical practice of the physiotherapists.

Finally, it is worth noting that only two trials [25,28], of the eleven trials analyzed in this systematic review performed the calculation to estimate the size of their samples. This led to a small total number of volunteers, of only 395 older adults in the eleven trials, which is very little, and makes it very difficult for the results of these studies to be generalized, being one more methodological problem found in the analyzed trials and which must be corrected by future trials on the topic.

### Biases related to the traits of the older adults

An important bias identified in the trials was the disproportionate age range of the CDOAs in the intervention and control groups. Only two of the studies showed no difference in the average age of the CDOAs [25,31], while five trials [21,22,24,26,30] demonstrated a difference, with average age variations between 1–8 years between one group and another, and the intervention group (aquatic physical therapy exercises) always exhibiting the youngest age groups.

This difference between the average ages of the intervention and control groups creates confounding bias in the studies, since at the end of the trial it can be questioned whether the outcomes analyzed improved in the intervention group by the aquatic physical therapy exercises or if this occurred because the participants were younger. This is a relevant question, since several studies have found that balance and gait worsen, favoring falls, with an increase in age in older individuals [52–63].

Another possible bias in the trials analyzed was the presence of sensory disorders in the older adults, such as hearing loss and vestibular dysfunctions, which have been widely associated with aging [64–67]. The trials did not report whether the samples exhibited these disorders, which were not exclusion criteria for any of the trials. This information is valuable, because several studies have reported that sensorineural hearing loss is a frequent finding in older people [68–70], and that older adults with hearing loss have worse balance, limited mobility and a greater likelihood of falls [71–79].

These balance and gait problems are also observed in children, adolescents and adults with hearing loss [80–89], suggesting that hearing loss may have a negative effect on balance and gait at any age. On the other hand, the use of hearing aids and cochlear implants increases auditory capacity and has improved the balance and gait, and reduced the risk of falls in older people with hearing loss [90–99], possibly due to the new auditory opportunities provided by these devices, suggesting that hearing input is not neutral in balance and motor skills [100,101].

This improvement in the balance of older adults with the help of hearing aids or cochlear implants may be justified, suggesting that sound signals transmitted to these individuals serve as fixed environmental reference points [102–104], providing spatial maps of the environment and better space-time orientation and balance [104,105]. This raises the question of whether it might be the moment to break paradigms and include hearing as another sensory system responsible for regulating human body balance [106].

Another uncertainty in the trial samples, also related to hearing loss and aging [107,108], is the presence of older people with vestibular dysfunction. This information is important since in some cases, one of the inclusion criteria for the older adults was to have a history of falls in the last six months, and vestibular dysfunction is the main cause of falls in the older adults, in addition to being a frequent finding in this population [109,120]. Balance and gait are altered in older people with vestibular dysfunctions, making them more susceptible to falls [121–133]. Thus, the presence of older individuals with hearing loss and/or vestibular dysfunctions in the trial samples analyzed would cause further confounding bias and may lead to underestimating the effect size of interventions, due to the greater balance and gait difficulty experienced by these individuals.

The difference between the average ages of the intervention and control groups, and the presence of the hearing loss and vestibular dysfunction in CDOAs are biases that should be controlled in future trials, since without hearing loss or vestibular dysfunction, the young old may obtain satisfactory results more rapidly, given their better balance and gait performance.

On the other hand, with hearing loss and vestibular dysfunction, old-old adults may demonstrate positive results later, because their balance and gait are more compromised. Thus, CDOAs can require different time periods to exhibit satisfactory results in balance, gait, quality of life and reducing fall-related outcomes. Age group, and the presence of the hearing loss and vestibular dysfunction may be serious confounding biases for future studies on the topic.

A suggestion for future trials, in order to avoid these biases, is block randomization according to older adults from each decade. In addition, older individuals with and without hearing loss and vestibular dysfunction should be randomized to make the groups more homogeneous in terms of age and the presence of hearing loss and vestibular dysfunction [134–136].

Thus, at the end of the trial, will be able to perform subgroup-analyses, in order to observe the effect of interventions in CDOAs in relation to age range, presence or absence of hearing loss and vestibular dysfunction, and determine whether the interventions were effective in improving balance, gait, quality of life and reducing fall-related outcomes in CDOAs of all age groups with and without hearing loss and vestibular dysfunction and thereby help guide clinical practice on the topic.

It is important to underscore that the difference between the age ranges of the CDOAs was a confounding bias included in the analysis of risk of bias and quality of the evidence in this review. The presence of hearing loss and vestibular dysfunction was not included, since we are uncertain whether these older adults were part of the trials, but we opted to mention the effect of hearing loss and vestibular dysfunction on the balance of older people, so that future trials on the topic can control these outcomes in their samples.

### Effectiveness of the aquatic physical therapy exercises in improving balance and gait speed (functional mobility) of healthy community-dwelling older adults (meta-analyses)

Meta-analyses on balance demonstrated that engaging in aquatic physical therapy exercises increases the functional reach, through of the anterior displacement of the center of pressure of CDOAs by 6.36cm, compared to their land-based counterparts, assessed by the FRT (p<0.00001), while the meta-analysis evaluated by POMA scale did not demonstrate significant differences between performing or not performing aquatic physical therapy exercises (p = 0.05), based on low-quality evidence (Figs 4 and 5 and Table 4).

**Table 4:** Quality of evidence of the trials that used aquatic physical therapy exercises to improve balance and functional mobility (gait speed) of community-dwelling older adults (meta-analysis).

Quality assessment							∖ of patients		Effect		Quality	Importance
∖ of studies	Study design	Risk of bias	Inconsistency	Indirectness	Imprecision	Other considerations	Aquatic group	Land-based or control group	Relative (95% CI)	Absolute (95% CI)		
**Balance (Anterior displacement of the pressure center):** (follow up: mean 8 weeks; assessed with: Functional Reach Test)												
02 [28,29]	RCT	very serious ^b,c,d,e^	not serious	not serious	not serious	none	26	31	-	**6.36** (5.22 to 7.50)	⨁⨁◯◯ LOW	CRITICAL
**Balance (Balance-related functional tasks):** (follow up: mean 13 weeks; assessed with: Performance Oriented Mobility Assessment Scale)												
02 [22,31]	QRT	very serious ^a,b,c,d,e^	not serious	not serious	not serious	none	37	39	-	**2.92** (-0.02 to 5.86)	⨁⨁◯◯ LOW	CRITICAL
**Gait (Gait Speed):** (follow up: 9 weeks; Timed Up and Go Test)												
03 [26–28]	RCT	very serious ^b,c,d,e^	not serious	not serious	not serious	none	58	57	-	**-0.29** (-0.61 to 0.02)	⨁⨁◯◯ LOW	CRITICAL

RCT: Randomized controlled trial; QRT: Quasi-randomized trial; a: There was no random sequence generation; b: No allocation secrecy; c: There was no blinding of the evaluator of outcome; d: Loss or incomplete data without performing the intention-to-treat analysis; e: Comparison of older adults’ groups with disproportionate age groups.

There were no significant differences between performing aquatic or land-based physical therapy exercises to improve functional mobility (gait speed) in the CDOAs (p = 0.07), measured by TUG, based on low-quality evidence, according Fig 6 and Table 4. It is important to emphasize that the TUG score is inversely proportional, such that the less time needed to complete the test, the faster the functional mobility (gait speed) of older adults.

These data are valuable for physiotherapists, since they show a clinical improvement in the balance assessed by the FRT and a trend towards improvement (as the black diamond touches the line of nullity) in the balance and gait speed of CDOAs, assessed, respectively, by the POMA Scale and the TUG, promoted by aquatic physical therapy exercises. These results guiding clinical practice and making it possible to analyze the risk of falling in this population after these interventions.

The literature contains several studies able to predict falls in CDOAs, based on the cutoff point of balance and gait assessment instruments [137–153]. Despite the differences in cutoff points, which indicate the fall prediction of these studies, we decided to discuss fall prediction in the older participants of this review, based on the averages obtained in meta-analyses and thereby identify which physical therapy exercises (aquatic or land-based) are more effective in decreasing the risk of falls in CDOAs.

A recent systematic review [154] found that the average of untrained CDOAs in the FRT was 26.6cm (CI:25.1 to 28.0). Another investigation reported that untrained CDOAs with a history of falling as assessed by the FRT obtained an average of 14.7cm [155]. The meta-analysis on the FRT demonstrated that aquatic or land-based physical therapy exercises resulted in averages above 26.6cm, showing the efficacy of the two physical therapy interventions in improving the balance of CDOAs. However, those who engaged in aquatic physical therapy exercises displayed a clinical improvement 6.36cm higher than those submitted to land-based exercises, demonstrating the greater efficacy of the former in improving the functional reach, through of the anterior displacement of the center of pressure of CDOAs and reducing their risk of falls.

We found no literature article with normative data on POMA scale values in CDOAs, or any cutoff point to predict falls, which hindered discussing meta-analysis data from the POMA scale. In addition, the POMA scale isolated seems not to be a consistent instrument for predicting the risk of falls in CDOAs [156].

Performing aquatic physical therapy exercises showed a downward trend the TUG time by 0.29 seconds, increasing functional mobility (gait speed) in CDOAs, compared to land-based physical therapy exercises. Literature reports differ in their cutoff point to predict the risk of falls in CDOAs assessed by the TUG, ranging between 8.5 and 20 seconds [157–163]. The highest agreement in cutoff points of the studies was between 11 and 12.4 seconds [164–167], a value we used to predict the risk of falls in CDOAs, related to the gait speed of this review.

The meta-analysis on functional mobility (gait speed) assessed by the TUG showed that both physical therapy interventions resulted in averages below 12.4 seconds in CDOAs, which demonstrated the efficacy of both physical therapy interventions in reducing falls in this population. However, in one of the trials [26], the post-test average of the land-based physical therapy exercise group was above 12.4 seconds, showing that aquatic physical therapy exercises promoted an even higher clinical improvement in functional mobility (gait speed) of CDOAs and a decline in their risk of falling.

Despite these findings, the evidence quality was low for all outcomes. Due to this low evidence quality of the trials involved in the meta-analyses, these findings should be interpreted with caution, because the estimate of the effect could be better proven, or refuted by future trials that present better methodological quality, according to the classification of the GRADE approach [20].

It is important to underscore that older adults with vestibulopathy exhibit slower gait speed [168] and that lower TUG (>11.1 seconds) and DGI scores (≤18 points) were correlated with falls in this population [169]. Thus, the average TUG meta-analysis scores indicate that aquatic physical therapy exercises can also be effective in improving balance, gait, and reducing the risk of falls in older adults with vestibular dysfunctions, since in two of the three trials, post-test averages were less than 11.1 seconds.

In addition to analyzing risk of falls, the TUG has also been used to observe the association between functional mobility (gait speed) and activities of daily living, morbidity and mortality in CDOAs [170–173], demonstrating also potential to quantify the effectiveness of the vestibular physical therapy [174], outcomes that can be analyzed in future trials on the topic.

### Other considerations

Despite the limitations and biases discussed above, the trials analyzed here exhibit positive characteristics, which should be mentioned and maintained in future trials. Ten trials described the physical therapy exercises used in the interventions (aquatic and land-based), except for Douris et al [24]. This information is very valuable, since it may help guide aquatic and land-based physical therapy exercise prescription to improve balance, gait, quality of life, and reduce fear of falling in CDOAs, making it possible to replicate these exercises in future trials and clinical practice (Appendix 2).

Another aspect that can be considered in future trials is to use more sensitive instruments to assess outcomes, such as dynamic computerized posturography or force platforms to evaluate balance; and accelerometers, software and camcorders to assess gait, in addition to validated clinical tests and scales. It is important to emphasize that there are normative data that predict the risk of falls for this population using these instruments, which can be applied in future trials to observe the efficacy of interventions in improving balance and gait and reducing falls in CDOAs [175–178].

In addition, future trials that use clinical tests or scales could also describe which motor tasks of these instruments the CDOAs had difficulty executing, since these tasks could receive greater attention and targeted rehabilitation to improve their performance in clinical practice.

The present systematic review found that aquatic physical therapy exercises were more effective than their land-based counterparts in improving balance, gait, quality of life, and reducing the fear of falling in CDOAs. During our literature search, we found several similar investigations, but these involved older adults with orthopedic or neurological disorders [179–184]. However, this review aimed at examining the issue from a health promotion and prevention standpoint, given that physiotherapists do not only work with rehabilitation. As such, this study provides data that confirm physiotherapists’ role in health promotion and prevention of age-related comorbidities.

An important gap observed in the trials analyzed was not including the otoneurological symptoms such as dizziness and vertigo, which are related to postural instabilities, imbalance and falls, commonly found in CDOAs and frequent in older adults with vestibulopathy. These outcomes could be included and analyzed in future trials in order to provide evidence on the effect of aquatic physical therapy exercises on the otoneurological symptoms of CDOAs, given that some evidences suggest positive effects of these exercises for individuals with vestibular hypofunction [15,16].

In addition, gaze stabilization exercises and otolithic repositioning maneuvers are considered key exercises for vestibular rehabilitation, demonstrating moderate-to-high evidence quality in reducing otoneurological symptoms and improving balance, gait, quality of life, and reducing falls in older adults with benign paroxysmal positional vertigo (BPPV) [185–195]. However, in addition to these exercises, there is a need for specific rehabilitation of the limits of stability, balance and gait, and therapeutic balance exercises have also demonstrated effectiveness in rehabilitating of the postural adjustments, providing better stability, balance and gait to older people with vestibular hypofunction [196–204].

As shown in this systematic review, aquatic physical therapy exercises were more effective in improving balance, gait, quality of life, and reducing the fear of falling in CDOAs. Thus, aquatic physical therapy may be a complementary therapy alternative for physiotherapists who also treat older individuals with vestibular hypofunction, in order to maintain the results achieved with balance exercises or vestibular rehabilitation maneuvers in older adults with BPPV and persistent residual dizziness.

Furthermore, aquatic physical therapy exercises may be an alternative for older adults concerned about performing land-based exercises, due to their comorbidities, fear of falling or postural instability, triggered by the complexity of the motor task [205–207], which may favor falls [208]. This shows the need for permanent physical therapy interventions for CDOAs, to improve these outcomes, since fear of falling is a common feeling in this population [209–218] and frequent in those with vestibulopathy [219–222], which may limit their movement and trigger other motor problems, such as kinesiophobia.

Finally, land-based physical therapy exercises also promoted a clinical improvement in the outcomes obtained by CDOAs and should be prioritized, given that gait, functional and leisure activities of daily living are performed on land. Thus, we believe that physical therapy treatment should include both exercises to promote more safety and stability for older adults, using aquatic physical therapy exercises when necessary, and a training program better suited to the individual’s reality, by prescribing land-based exercises when they exhibit greater body stability.

This systematic review observed that aquatic physical therapy exercises improved balance, gait, quality of life, and reduced fear of falling in CDOAs in trials in which the intervention program consisted of sessions lasting 30 minutes or longer, twice or more a week for at least five weeks [21–31]. These data may help guide future trials using aquatic physical therapy exercises for CDOAs and the clinical decision-making of physiotherapists.

The limitation of this systematic review was not searching in thesis and dissertation databases.

## Conclusion and implications for future trials

Aquatic physical therapy exercises were more effective than their land-based counterparts in improving balance, gait, quality of life, and reducing fear of falling in CDOAs. However, due to the low evidence quality, the results of the trials analyzed here should be interpreted with caution.

Low evidence quality on the topic and the lack of information in the trials on the presence or absence of adverse effects caused by the two interventions hindered analysis of the benefits and adverse effects of the interventions, meaning this clinical decision remains inconclusive. Any estimate presented by this review regarding a recommendation involving aquatic physical therapy exercises to improve balance, gait, quality of life and reduce fear of falling in CDOAs would be uncertain, inconclusive or insufficient.

Given the low evidence quality observed in this review, we suggest that future trials on the topic be proposed, with better methodological quality to promote high-quality evidence on the effectiveness of aquatic physical therapy exercises in order to improve balance, gait, quality of life, and reduce fall-related outcomes in CDOAs. Future trials should control biases related to sample selection, blinding of outcome assessors, sample losses, or conduct intention-to-treat analysis, control average participant age, the presence of hearing loss and vestibular dysfunction in the sample.

We also suggest that future trials observe, in addition to balance and gait, clinical, functional and otoneurological outcomes, such as dizziness, vertigo, functionality, performance in activities of daily living and leisure, also emphasizing the fall-related outcomes and the quality of life of older adults, since, as shown in the present review, only two of the eleven trials analyzed these outcomes.

Moreover, future trials could also investigate the costs of these interventions and include a follow-up period, in order to determine how long the effects of these interventions (aquatic and land-based) last in CDOAs. This information is valuable because it will provide theoretical-scientific support to guide physical therapy clinical practice on the topic and ensure that interventions are based on high-quality methodological evidence.

## Supporting information

S1 Checklist PRISMA checklist of items to include when reporting a systematic review involving a network meta-analysis. (DOCX)

S1 Appendix Search strategies. (DOCX)

S2 Appendix Description of the therapeutic exercises used in the interventions of the trials included in this systematic review. (DOCX)
