# Supplementary material for: Effectiveness of the aquatic physical therapy exercises to improve balance, gait, quality of life and reduce fall-related outcomes in healthy community-dwelling older adults: A systematic review and meta-analysis
Source: PLoS One. 2023 Sep 8;18(9):e0291193. doi: 10.1371/journal.pone.0291193 (PMC10490910; doi:10.1371/journal.pone.0291193)
Supplement: S1 Appendix — (DOCX) [file pone.0291193.s002.docx]

Effectiveness of the aquatic physical therapy exercises to improve balance, gait, quality of life and reduce fall-related outcomes in healthy community-dwelling older adults: A systematic review and meta-analysis.

**Appendix 1 - Search Strategies**

(aged[mh] OR aged[tiab] OR elderly[tiab] OR senior[tiab] OR Older adults OR senium[tiab]) AND (Hydrotherapy[mh] OR Hydrotherap*[tiab] OR "Bath, Whirlpool"[tiab] OR "Baths, Whirlpool"[tiab] OR "Whirlpool Bath"[tiab] OR "Whirlpool Baths"[tiab] OR "kneipp therapy"[tiab] OR "kneipp treatment"[tiab] OR "water immersion therapy"[tiab] OR (("Physical Therapy"[tiab] OR "Physical Therapies"[tiab]) AND (marine[tiab] OR water[tiab] OR aquatic*[tiab]))) AND ("Postural Balance"[mh] OR "body equilibrium"[tiab] OR "body sway"[tiab] OR "equilibrium, body"[tiab] OR "musculoskeletal equilibrium"[tiab] OR "postural balance"[tiab] OR "postural equilibrium"[tiab] OR gait[mh] OR gait[tiab] OR Dizziness[mh] OR dizziness[tiab] OR "giddiness"[tiab] OR "light headedness"[tiab] OR "lightheadedness"[tiab] OR Vertigo[mh] OR Vertigo[tiab] OR walking[mh] OR walking[tiab] OR Syncope[mh] OR fainting[tiab] OR faintness[tiab] OR lipothymia[tiab] OR syncope[tiab] OR "vasovagal attack"[tiab] OR "vasovagal collapse"[tiab] OR "vasovagal shock"[tiab] OR "vasovagal syncope"[tiab] OR ((otoneurologic*[tiab]) AND (syndrome*[tiab] OR outcome*[tiab])))
