# Supplementary material for: Effectiveness of the aquatic physical therapy exercises to improve balance, gait, quality of life and reduce fall-related outcomes in healthy community-dwelling older adults: A systematic review and meta-analysis
Source: PLoS One. 2023 Sep 8;18(9):e0291193. doi: 10.1371/journal.pone.0291193 (PMC10490910; doi:10.1371/journal.pone.0291193)
Supplement: S2 Appendix — (DOCX) [file pone.0291193.s003.docx]

**Appendix 2.** Description of the therapeutic exercises used in the interventions of the trials included in this systematic review.

| **Authors** | **Control group** | **Intervention group** |
| --- | --- | --- |
| **Avelar et al[21]** | **Phase I - warming up**  Walking: gait with progressive speed, up to 3 minutes.  Stretching (the stretching positions were sustained for 30 seconds):  Stretching of the hamstring muscles  Position in the pool and on the floor: orthostatic position with the back resting against the wall.  Activity in the pool: to lift one of the lower limbs, while maintaining knee extension and ankle dorsiflexion.  Activity on the floor: to perform spinal flexion, while keeping the lower limbs stretched out.  Stretching of the rectus femoris and iliopsoas muscles  Position in the pool and on the floor: orthostatic position with both hands resting on the edge of the pool or against the wall, respectively.  Activity in the pool and on the floor: to perform knee flexion, sustaining it with the aid of the ipsilateral upper limb, in association with hip extension.  **Phase II - muscle endurance exercises**  Exercise 1: endurance exercise for the anterior muscles of the thigh  Position in the pool and on the floor: orthostatic position with the back resting against the wall.  Activity in the pool and on land: to perform hip flexion with knee extension (4x20).  Exercise 2: endurance exercise for the posterior muscles of the thigh  Position in the pool and on the floor: orthostatic position with the hands resting on the edge of the pool, or against the wall, respectively.  Activity in the pool and on the floor: to perform hip flexion with knee extension, while keeping the spine straight (4x20).  Exercise 3: endurance exercise for the lateral muscles of the thigh  Position in the pool and on the floor: orthostatic position, perpendicular to the supporting edge, with the hands resting on the edge of the pool or wall, respectively.  Activity in the pool and on the floor: to perform hip abduction, while avoiding any spinal movement (4x20).  Exercise 4: endurance exercise for the medial muscles of the thigh  Position in the pool and on the floor: orthostatic position, perpendicular to the supporting edge, with the hands resting on the edge of the pool or wall, respectively.  Activity in the pool: to perform adduction (return from the abduction) of the hip, while avoiding any spinal movement (4x20).  Activity on the floor: to perform adduction beyond the median line, while preventing the spine from moving (4x20).  Exercise 5: endurance exercise for triple flexion of the lower limbs  Position in the pool and on the floor: orthostatic position with the back resting against the wall.  Activity in the pool and on the floor: to perform triple flexion of the hips, knees and ankles (4x20).  Exercise 6: endurance exercise for the plantar flexors  Position in the pool and on the floor: orthostatic position facing the edge of the pool or wall, respectively, with support only in the event of lack of balance.  Activity in the pool and on the floor: plantar flexion in association with knee extension (4x20).  Exercise 7: endurance exercise for dorsiflexors  Activity in the pool and on the floor: to perform gait while standing on heels (three series lasting one minute each with a 30-second interval between them).  **Phase III - cooling down**  Walking  Activity in the pool and on the floor: gait with regressive speed, for up to three minutes. | |
| **Bruni et al[22]** | No intervention | The aquatic physical therapy sessions had the following activities: walks in the pool, strengthening and stretching  lower limb muscles and activities for postural balance training. |
| **Cunha et al[23]** | No intervention | The aquatic physical therapy exercises were developed through extensive research, being divided into three subdivisions: stretching the biceps brachii muscle; horizontal abduction and adduction with wand; stretching the triceps brachii muscle; of the anterior muscles of the leg; calf muscles, tretching the quadriceps muscle; exercise for strength training abduction and horizontal adduction; resistive; rowing; outstretched legs beat; jump with distance; relaxation; stretching: here lateral and neck musculature stretching were used. |
| **Douris et al[24]** | The exercises used in each of the groups were not specified. | |
| **Elbar et al[25]** | No intervention for 16 weeks, and then they performed the same aquatic exercise program as the intervention group. | Sessions were led by one licensed physical therapist and assisted by one licensed hydrotherapies. The aquatic physical therapy program was performed at five progressively more challenging levels of difficulty with respect to strength and balance function. While subjects stand in the water and maintain a stable upright stance over the base of support (BOS), water movement and turbulence overloading the postural control systems during standing and reaching movement (while feet are fixed on the pools floor) and during change of support movement (e.g., stepping). For standing exercise (levels 1 and level 2 exercises), this relative motion of water causing displacement of either the body’s Center of Mass (COM) (via water motion and turbulence) or the BOS (standing on unstable balls or a ‘‘noodle’’ placed underneath the subject’s feet on the pool floor), these exercises in water might cause perturbed balance (e.g., during leaning, turning, reaching) thus challenging the balance control system. During gait exercises (exercise levels 3 and 4), the water can create disturbance due to water resistance motion and turbulence act to perturb the COM (e.g., simulate tripping) and BOS perturbations (e.g., simulate slipping due to a slip on the pools floor). Additional exercises (level 5) such as perturbation exercises were provided by the instructors or classmates to evoke balance recovery stepping reactions against the water resistance. The perturbation methods may fulfill the fundamental biomechanical requirement (disruption of the COM–BOS relationship) and elicit postural and stepping reactions that are similar in many respects to land-based training. Hence, it is possible that the training benefits derived using water-based training that includes perturbation exercises may generalize to the reactions evoked outside the water. It is important to note that exercises were adjusted by the therapist throughout the program to match each subject’s ability and to be continuously challenging but never dangerous. The goal of each session in the current program is to constantly challenge the postural control system and stepping responses with exercises. Training programs should be tailored to the individual needs and abilities of the participant. This process requires interaction with the instructors and was not based on a ‘‘cookbook’’ approach to training. As in any type of training, for improvement to occur it is crucial to maintain a progressive and specific training load for each of the participants.  Progressions were made when the individuals have reached adaptation. On each level the instructor modified an exercise to be more or less challenging for each participant. During all the exercises, the instructor and the lifeguard were nearby and fully alert to the security of each participant. |
| **Franciulli et al[26]** | Exercise 1: Breathing control. Positioning: sitting on the Swiss ball. Shoulders in 90º flexion and elbows in extension, being associated with a trunk extension. Activity: inspiration through the nose and expiration through the mouth in a slow and prolonged manner, maintained for 2 seconds (2 minutes);  Exercise 2: Stretching the hamstring muscles. Positioning: orthostatic position with the back against the wall. Activity: elevate one of the lower limbs, maintain knee extension and ankle dorsiflexion with thera band (3 sets of 30 seconds).  Exercise 3: Stretching the triceps surae and iliopsoas muscles. Positioning: orthostatic position with hands resting on the wall. Activity: take a large step forward, keep the anterior knee in flexion, the posterior knee in extension and the feet in contact with the floor (3 sets of 30 seconds).  Exercise 4: Bilateral shoulder horizontal abduction-adduction. Positioning: semi-sitting position, shoulders flexed at 90º, elbows extended. Activity: start in adduction up to 90º of horizontal abduction (3 sets of 10 repetitions).  Exercise 5: gait with stops in single-leg stance. Activity: walk and, at the command of the physical therapist, maintain unipedal support with the opposite knee in flexion for 10 seconds (12 stops).  Exercise 6: Chest expansion. Positioning: individual in dorsal decubitus, with knees bent and feet supported, upper limbs also supported on the stretcher. Activity: start the movement with the elbows together in front of the face, abduct and laterally rotate the shoulders with inspiration until your hands are facing the ceiling, and exhaling while the elbows are brought closer together (10 repetitions).  Exercise 7: Strengthening the thoracic erectors. Positioning: subject in ventral decubitus, holding the side of the stretcher bilaterally. Activity: the individual extends the arms in a way that supports their upper trunk, performing cervical extension, instructing them to inhale and expand the rib cage (2 sets of 10 seconds of support).  Exercise 8: Balance training on an unstable surface. Activity: individual trains balance on balance board.  Exercise 9: Walking with obstacles. Activity: individual trains gait by passing through various obstacles on the ground. | Exercise 1: Stretching the hamstring muscles. Positioning: orthostatic position with the back against the wall. Activity: elevate one of the lower limbs, maintain knee extension and ankle dorsiflexion (3 sets of 1 minute).  Exercise 2: Stretching the triceps surae and iliopsoas muscles. Positioning: orthostatic position with hands resting on the edge of the pool. Activity: take a large step forward, keep the anterior knee in flexion, the posterior knee in extension and the feet in contact with the bottom of the pool (3 sets of 1 minute).  Exercise 3: Bilateral shoulder flexion-extension. Positioning: orthostatic position. Activity: perform shoulder flexion and extension keeping elbows in extension. Associating inspiration in flexion and expiration in extension. Start in maximal hyperextension of the shoulders until flexion at 90º (12 repetitions).  Exercise 4: Bilateral shoulder horizontal abduction-adduction. Positioning: semi-sitting position, shoulders flexed at 90º, elbows extended. Associating inspiration with abduction and expiration with adduction. Activity: start in adduction up to 90º of horizontal abduction (12 repetitions).  Exercise 5: Breathing control. Positioning: semi-sitting position without posterior support, with immersion at shoulder level. Shoulders in 90º flexion and elbows in extension. Associated with a trunk extension. Activity: slow and prolonged expiration through the mouth over the water, with the mouth immersed and later with the mouth and nose immersed (6 repetitions each).  Exercise 6: Walking in a Row. Positioning: place your hands on the shoulder of the individual in front. Activity: move around the pool making curves and changes in direction and levels. Activity led by the physical therapist (5 minutes).  Exercise 7: Walking with stops in single-leg stance; Activity: walk and, at the command of the physical therapist, maintain unipedal support with the opposite knee in flexion for 10 seconds (12 stops).  Exercise 8: Walking with one foot in front of the other. Activity: marching with one foot immediately in front of the other, and so on (6 repetitions).  Exercise 9: Ankle Pumping. Positioning: orthostatic posture and trunk extension. Activity: perform knee extension associated with plantar flexion, maintain position for 5 seconds and then knee flexion associated with dorsiflexion, also maintaining for 5 seconds (3 sets of 10 repetitions). |
| **Oh et al[27]** | The exercises for both groups involved the same muscular groups, and the rating of perceived exertion was maintained at 4 of a 10-point scale (i.e., somewhat heavy exertion). All the instructors had more than 3 years of clinical experience in physical therapy. Each exercise was conducted by one instructor and two assistant instructors. The total exercise time was 60 min. The participants performed stretching outside of the pool for 5 min. Warm-ups consisted of walking in the pool for 5 min, and the main exercises lasted 40 min. During the last 10 min, all the groups were guided through a cool-down and breathing routine. The exercises used were: Flexibility exercise: Gently shoulder abduction & adduction with knee flexion; shoulder abduction & Toe kick with knee extension: front, back, left, right strength and endurance exercise; One leg standing with eye open or closed; One leg standing, Shoulder abduction with hip flexion, extension, abduction, adduction; Single leg hopping, double leg jumping  Cool-down (10 min); Walking forward, backward, sideward stretching. | |
| **Silva et al[28]** | The aquatic physical therapy exercises were: Gait (Front and lateral gait); Gait with direction changes; Gait with obstacles; Double tasking. Transfer training through Halliwick rotation controls (transversal, sagittal, longitudinal and combined) and Strength training through the Bad Ragaz ring method (Trunk, upper and lower limb patterns). | The Land-based physical therapy exercises were: Gait (Front and lateral gait); Gait with direction changes; Gait with obstacles; Double tasking. Transfer training (Sit-to-Stand training, decubitus changes and reaching the object to the front); Strength training through the proprioceptive neuromuscular facilitation technique (Trunk, upper and lower limb patterns) and balance training. |
| **Simmons et al[29]** | Exercise tasks — land and water groups. — The exercise regime, done to subject tolerance with rest periods as needed, was identical for the water and land exercise groups. The specific exercises were: Walking forward and backward; walking backward while high stepping, marching forward and backward with knees bent; walking forward and backward with knees straight; sidestepping without crossing the legs; sidestepping with crossing the legs; heel-to-toe walking forward and backward; marching in place; standing partial squats; toe raises; heel raises; kicking in a diagonal; kicking in cardinal planes of motion and twisting. | |
| **Tavares et al[30]** | The land-based intervention protocol was performed through warm-up, stretching, balance training and stretching. The warm-up consisted of walking forward with large steps and alternating movements of shoulder flexion at 90° (1 minute); walking forward with 90º hip and knee flexion, alternating upper limb movements (1 minute); walking sideways with large steps associated with lateral movement of the upper limbs (1 minute for each side); play ball with the therapist (1 minute). In stretching, exercises were performed with knee extensors (1 on each side), hip extensors (1 minute on each side) and trunk extensors (1 minute on each side). During strengthening (1 minute and 15 seconds on each side) hip extensors, upper trunk extensors, knee extensors and lower trunk extensors were used. During the Balance Training (2 minutes and 30 seconds each exercise in which the instability was performed by the hip) the individual remained seated on the ball without bringing the feet close to the ball and or supporting the hands on the ball or thighs, so that they did not if fixed; exercise with the balance board; rambling over spaghetti; exercise with anterior oscillation performed by the physical therapist; exercise with posterior oscillation performed by the physical therapist, with forearm support in the anterior and posterior region of the patient's trunk; exercise with mini-tramping in orthostatism with unilateral support; standing exercise with hindfoot support, mini-tramping exercise playing ball with the physical therapist; exercise on the rocker. Finally, during relaxation (1 min each exercise) cervical traction, lumbar traction, friction, Swiss ball massage were performed. | The aquatic intervention protocol was also performed through heating, stretching, strengthening and relaxation. During the warm-up, exercises were performed: walking forward (1 minute) and then back (1 minute) with large steps and anteroposterior movement of the upper limbs on the water surface with 2 small dumbbells, walking sideways with large steps and with lateral movement of the upper limbs (1 minute for each side), walking forward with 90º flexion of the hip, knee and ankle, alternating movement of the upper limbs, in dissociation of the waists (1 minute). In the Stretching phase (2 minutes of each exercise) the hip, knee and trunk extensors were stretched. Strengthening focused on the knee extensors, upper trunk, lower trunk and hip muscles (2 minutes and 30 seconds each exercise). During Balance Training (2 minutes and 30 seconds each exercise in which the instability was performed by the physical therapist turbulence sometimes in the anterior region, sometimes in the posterior region or even on the sides, having to return to the midline) sitting on the ball with triple flexion of the lower limbs standing on one of the limbs and semi-flexed knees; sitting facing forward with the physical therapist's support in the scapula or hamstring region without support from the feet to the floor with oscillations to the sides; sitting on the back with the physical therapist's support in the palm region without support from the feet to the floor with oscillations to the sides; standing on planks performing hip flexion-extension; standing on boards performing abduction-adduction; standing on the proprioception board; standing with posterior oscillation by the patient's trunk keeping the weight only on the heels; Finally, the Relaxation exercises (1 minute each exercise with the patient in a supine position with floats on the pelvic girdle and on the heels) were performed with the patient receiving support in the occipital region and cervical inclinations like a snake; anteropulsion and trunk retropulsion movement as a wave; cervical traction during patient expiration; receives turbulence throughout the posterior trunk region. |
| **Vale et al[31]** | For control group, there was no intervention. The participants only performed the physical-functional evaluations at the beginning of the study, and after four months. After that the end of the present study, the control group participants were invited to start the aquatic program (the same protocol offered to intervention group). | The exercise program provided 29 motor activities: 6 were warm-up activities, 8 muscle strengthening exercises, 11 flexibility training activities, and 4 relaxation activities. The exercises (the use of floats for resistance and the number of repetitions) varied according to the degree of difficulty, determined by group performance. |
